# Beyond survival: the lasting effects of premature birth

**DOI:** 10.3389/fped.2023.1213243

**Published:** 2023-07-07

**Authors:** Daniela Morniroli, Valentina Tiraferri, Giulia Maiocco, Domenico Umberto De Rose, Francesco Cresi, Alessandra Coscia, Fabio Mosca, Maria Lorella Giannì

**Affiliations:** ^1^ Department of Clinical Sciences and Community Health, University of Milan, Milan, Italy; ^2^ Neonatology of the University, Department of Public Health and Pediatric Sciences, University of Turin, Turin, Italy; ^3^ City of Health and Science of Turin, Turin, Italy; ^4^ Neonatal Intensive Care Unit, “Bambino Gesù” Children’s Hospital IRCCS, Rome, Italy; ^5^ Neonatal Intensive Care Unit (NICU), Fondazione IRCCS Ca’ Granda Ospedale Maggiore Policlinico, Milan, Italy

**Keywords:** preterm infant, growth, human milk, outcomes, adult age

## Abstract

Preterm birth, defined as birth before 37 weeks of gestation, is a major public health concern. It affects about 10% of all newborns globally and is the main cause of infant death and morbidity. Prematurity increases the likelihood of respiratory distress syndrome, cerebral palsy, and developmental abnormalities. Furthermore, premature newborns are at risk of acquiring chronic noncommunicable diseases later in life due to interference with organ system development during the in-utero and perinatal period. Because of the greater risk of long-term repercussions, preterm birth should be considered a chronic disorder, and gestational age and other birth histories should be included in all medical records for patients of all ages, especially when assessing the risk of multiple chronic diseases. Conventional methods for assessing preterm infant development, as well as reliable and precise growth monitoring, can lead to the early detection of growth decline and the adjustment of feeding regimens as needed. Because of its unique composition and useful components, human milk is a powerful tool for mitigating the negative outcomes associated with prematurity. It contains a variety of growth factors that promote the development of organs and systems, counteracting the negative effects of the abrupt interruption of intrauterine development and promoting better outcomes in all altered functions. Despite its multiple benefits, human milk cannot totally restore the lasting damage caused by premature birth. Premature infants cannot be completely overcome by nutrition alone, and yet adequate nutritional intake and human milk feeding are critical to their health and development.

## 1. Introduction

Preterm birth is defined by the World Health Organization (WHO) as a birth that occurs before 37 completed weeks of gestation (1). It is a significant public health concern, accounting for an estimated 10% of births worldwide (2), with approximately 1 in 10 babies born prematurely in 2019 (3). Preterm birth is the leading cause of infant mortality and morbidity, and it is associated with an increased risk of respiratory distress syndrome, cerebral palsy, and developmental delays.

Identifying risk factors and implementing interventions to prevent premature birth and improve outcomes for premature infants is crucial. The risk of having long-term negative outcome increases as gestational age at birth decreases. 54.6% of adults (18–64 years old) born preterm do not develop comorbidities; this percentage falls to 23 percent if we consider adults born before 28 weeks of gestation (4). It is now evident that low gestational age at birth is associated with an increased risk of developing chronic non-communicable diseases, which account for the majority of the leading causes of death in adulthood (5). Preterm birth has also been associated with an increased risk of mental health issues such as depression, anxiety, and Attention Deficit-Hyperactivity Disorder (ADHD) (6).

Also, preterm infants may experience difficulties in achieving optimal growth, and they frequently suffer from extrauterine growth restriction (7), which can cause long-term health issues(8).

Monitoring and promoting healthy growth is necessary to improve the long-term outcomes of preterm infants.

## 2. Long-term effects of premature birth

There are numerous long-term medical, educational, and social consequences associated with preterm birth (Table 1). Preterm infants frequently experience respiratory distress syndrome (RDS). RDS occurs when preterm infants' lungs are underdeveloped and unable to produce enough surfactant, which helps keep the lungs open. RDS-affected preterm infants may need respiratory support, such as mechanical ventilation or non-invasive respiratory support, to help them breathe (9, 10). Necrotizing enterocolitis (NEC), which inflames and damages the intestines, may also affect premature infants (11). NEC is a severe condition that can lead to perforation of the intestine and sepsis, a potentially fatal infection (11). In addition, preterm infants are at a higher risk for several neurological disorders, including cerebral palsy, which affects movement and coordination, and developmental delays (12). Premature infants also have an increased risk for intraventricular haemorrhage (IVH), a type of brain bleeding that can result in neurological complications (13). Compared to term-born infants, preterm infants are more likely to develop cognitive deficits, such as lower intelligence quotient (IQ) scores and academic performance (14). These deficits may continue through adolescence and adulthood (15).

**Table 1:** Poor outcomes caused by prematurity and described in the literature.

Poor outcomes related to prematurity	
Respiratory issues	• Respiratory Distress Syndrome • Bronchopulmonary dysplasia • Higher risk of recurrent hospitalizations due to respiratory/infectious disease (such as bronchiolitis)
Cardiovascular issues	• Increased risk of heart failure and ischemic heart disease • Increased risk of higher systolic and diastolic pressure
Gastrointestinal issues	• Poor feeding tolerance • Necrotizing enterocolitis • Short bowel syndrome
Metabolic issues	• Higher and altered adiposity • Lipid disorders • Increased risk of higher insulin resistance and diabetes mellitus type 2 • Increased risk of metabolic syndrome
Kidney issues	• Chronic kidney disease • Low nephron number
Growth issues	• Extrauterine growth restriction • Failure to thrive • Impaired Catch-up growth
Neurological and cognitive issues	• Motor delay (both fine motor and gross motor delays) • Cerebral palsy • Cognitive impairment with lower intelligence quotient scores and academic performances in several areas
Mental health issues and social interactions	• Internalising problems (eg, anxiety, depression) • Attention Deficit—Hyperactivity Disorder (ADHD) • Autism spectrum disorder • Social issues, with a tendency to be shyer, have fewer friends, be more socially withdrawn, and be more likely to be bullied than term born peers

Additionally, preterm infants are more likely to develop behavioural issues, such as ADHD and anxiety disorders (16). These issues may have significant long-term repercussions, including poor academic performance and social isolation. Compared to term-born infants, preterm infants are more likely to struggle academically, as evidenced by lower grades and higher rates of grade retention (17). These difficulties can persist into adulthood, negatively impacting employment and overall quality of life.

Prematurity is associated with the development of chronic conditions and multimorbidity. Individuals born at an early gestational age are exposed to in-utero and perinatal events that can interfere with organ system development at critical stages. Lungs, kidneys, brain, and heart reach full maturity during the last trimester of pregnancy, when intrauterine and early postnatal environments may permanently alter organ structure and metabolism, thereby programming for chronic disease in adulthood. Several pathways influence organ development. The maturation of intrauterine organs is influenced by maternal and paternal genetic patterns, adverse maternal health conditions, pregnancy complications, and pathological placental conditions that can impede intrauterine growth or lead to preterm birth. Postnatal events, such as perinatal inflammation, clinical complications of premature birth, and unintended consequences resulting from intensive care and after-discharge care practices, can also influence the outcome. Adults born prematurely are at risk for developing kidney diseases such as chronic kidney disease and hypertension (18–20). Normally, the number of nephrons increases proportionally with birth weight and gestational age. 60% of nephrons are formed during the third semester, which lasts until week 36 of pregnancy. No nephrons develop after birth. This means that preterm newborns have quantitative and qualitative alterations in nephron formation. Reduction of total nephrons increases susceptibility to hypertension due to decreased sodium excretory capacity and chronic kidney disease (CKD) due to compensatory hyperfiltration and nephron hypertrophy (21, 22). Moreover, preterm birth has been associated with an increased risk of cardiovascular disorders. In the third trimester, cardiomyocyte hyperplasia enables rapid cardiac growth. The growth of cardiomyocytes changes from foetal hyperplasia to neonatal hyperplasia after birth. This switch occurs too early in prematurely born individuals, resulting in morphological and functional cardiac impairments and an increased risk of heart failure and ischemic heart disease. Preterm-born adults have a smaller biventricular chamber and a diminished cardiac reserve, which may result in systolic and diastolic dysfunction. The risk of cardiac dysfunction can also be caused by other preterm birth complications, such as bronchopulmonary dysplasia (BDP), which exacerbates right ventricle dysfunction, lower ventricle chamber size, and pulmonary hypertension. In addition, preterm birth may impede vasculature development due to low elastin synthesis, exposure to high oxygen levels, and low angiogenic capacity. Not only irreversible vascular structure and function alterations do increase the risk of developing hypertension and other cardiovascular disorders, but they also impair organogenesis, raising the risk of premature complications such as retinopathy of prematurity (ROP) and BPD (23). The risk of elevated blood pressure is even greater in preterm infants with a very low birth weight due to unfavorable adiposity, as their fetal development was impaired (24).

Clearly, preterm birth must be regarded as a chronic condition with an increased risk of long-term consequences. As a result, gestational age and other birth histories should be included in all medical records for patients of all ages, especially when assessing the risk of multiple chronic diseases, to improve the prevention and detection of adverse health events (5, 18, 25).

## 3. The significance of postnatal growth for health outcomes

Postnatal growth plays a crucial role in programming long-term health outcomes, especially in premature infants. Studies indicate that preterm infants who experience catch-up growth during the postnatal period have improved cognitive and motor outcomes and a decreased risk of developing chronic health problems (26). Appropriate postnatal growth is also essential for cognitive and neurodevelopmental outcomes. Preterm infants with poor postnatal growth have a higher risk of developmental delays and disabilities, as well as lower cognitive and academic achievement, compared to premature infants who underwent adequate postnatal growth (27). Premature infants frequently suffer from extrauterine growth retardation, a condition in which infants fail to reach their expected growth potential during the postnatal period (28). This condition can have significant long-term health consequences, including poor neurodevelopmental outcomes and an increased risk of chronic health issues. Several factors, such as inadequate nutrient intake, poor feeding tolerance, and increased metabolic demands, contribute to preterm infants' growth difficulties (29). In addition, medical conditions such as respiratory distress syndrome and necrotizing enterocolitis may contribute to inadequate postnatal growth. In light of this evidence, healthcare professionals must closely monitor the growth of preterm infants and take measures to ensure proper growth, including human milk feeding and nutritional interventions.

## 4. The importance of precise and accurate growth tracking

Understanding the health and developmental outcomes of premature infants requires a comprehensive approach to monitoring their growth. Accurate and precise growth monitoring can lead to the early detection of growth deterioration and allow for the modification of feeding strategies accordingly. Standard methods for evaluating preterm infants' growth include anthropometric measurements such as weight, length, and head circumference. These measurements provide vital information on the overall growth pattern and can be used to compare the infant's growth to established growth charts and benchmarks (30, 31).

However, anthropometric measurements alone may not provide a complete picture of a child's growth and development because they do not account for changes in body composition. Body composition measurements provide a more complete understanding of an infant's development by assessing the proportions of fat mass and fat-free mass. Methods such as dual-energy x-ray absorptiometry (DXA), air displacement plethysmography (ADP), and bioelectrical impedance analysis (BIA) are commonly used to measure the body composition of premature infants (32). These methods provide valuable insights into the quality of growth, enabling healthcare professionals to assess whether an infant is achieving the proper proportions of lean body mass and adipose tissue. Nonetheless, a growing body of research indicates that the accurate measurement of length and head circumference can provide reliable information on fat-free mass deposition and brain growth, respectively (33), highlighting the significance of a routine, standardized, weekly measurement of all auxological parameters to ensure a comprehensive evaluation of preterm growth. Accurate longitudinal monitoring of growth enables the early detection of growth retardation in premature infants as soon as possible during NICU stay, rather than a single cross-sectional evaluation typically assessed at discharge.

Growth retardation, characterized by failure to achieve expected growth rates, can result in negative health outcomes, such as impaired neurodevelopment and increased morbidity (34). The implementation of appropriate interventions reduces the risk of long-term complications when slowed growth is detected in a timely manner. Monitoring growth accurately provides valuable information to healthcare professionals, allowing them to tailor feeding strategies to meet the specific requirements of each preterm infant. Adaptations can be made to caloric intake, nutrient composition, and feeding methods based on the infant's growth trajectory and proximal body composition measurements, such as length and head circumference (35). This individualized approach to nutrition promotes optimal growth and development in premature infants, ultimately enhancing their health outcomes.

## 5. The effect of human milk feeding on preterm development: a modulator, but not a cure all toll

Human milk feeding is essential for promoting optimal growth and development in infants. It contains a unique combination of macronutrients, micronutrients, and essential developmental factors for infant growth and development (36). The American Academy of Pediatrics recommends exclusive breastfeeding for the first six months to ensure optimal health (37). Human milk contains numerous bioactive factors, including immunoglobulins, lactoferrin, lysozyme, and cytokines, which contribute to the infant's immune system and provide protection from infection (38). These factors are of paramount importance for preterm infants, who have immature immune systems and are more prone to infection. Human milk feeding has been linked to enhanced cognitive and neurodevelopmental outcomes in infants. Several studies have demonstrated that breastfed infants have higher cognitive scores, even after controlling for family environment and other confounding variables. The availability of human milk, particularly for very low birth weight (VLBW) infants, presents a significant challenge. In various healthcare settings, infants with VLBW may not have access to donor milk, which can hinder their development (39). Efforts to support and promote breastfeeding and donor milk programs are essential for addressing this issue. Human milk is the optimal source of nutrition for infants, but it may not meet the unique nutritional requirements of premature infants (40), who require careful monitoring of their growth and nutritional status to ensure that their specific needs are met. Therefore, it may be necessary to supplement human milk in this population to ensure proper growth and development. Emerging methods of individualized fortification promise in improving postnatal growth and neurodevelopmental outcomes in preterm infants (41).

Human milk is a potent tool for mitigating some of the negative outcomes associated with prematurity due to its unique composition and functional substances. Enhancing the development and function of preterm infants' immune systems is one of the most notable benefits of human milk for premature infants. Moreover, human milk contains numerous growth factors that promote the maturation of the gastrointestinal tract, thereby facilitating optimal nutrient absorption and digestion (42). Brain development is another essential function of human milk for premature infants. Long-chain polyunsaturated fatty acids, such as docosahexaenoic acid (DHA) and arachidonic acid (ARA), are essential for the development and maturation of the brain and nervous system (43). These fatty acids have been linked to enhanced cognitive and visual development in preterm infants, highlighting the significance of human milk to their overall health. The objective of the study by Finken et al. (44) was to determine the cardiovascular effects of exclusive breastfeeding in early childhood by examining the relationship between lipid profile, carotid intima-media thickness (CIMT), and body composition in young adults who were born extremely prematurely. The study found that preterm subjects had lower levels of high-density lipoprotein (HDL) cholesterol and a greater CIMT than the control group. Those preterm subjects with a higher lean mass had a lower CIMT, whereas those with a higher fat mass had a higher CIMT. These findings suggest that early growth and current body composition play a role in the lipid profile and cardiovascular health of very preterm young adults. In addition, a recent study (45) conducted a cross-sectional analysis of data collected from 347 children aged 9–13 years. Children who were breastfed for a longer duration had lower levels of total cholesterol, low-density lipoprotein (LDL) cholesterol, and triglycerides than those who were breastfed for a shorter duration or who were never breastfed. Furthermore, the study found that exclusive breastfeeding duration was inversely associated with cardiovascular disease risk factors (45). The findings of the study suggest that breastfeeding may protect against the development of CVD risk factors in children. These results support the recommendation to promote breastfeeding as the optimal method of infant feeding, given that it can have long-term health benefits for children.

## 6. Discussion

Preterm infants face a variety of health issues, including underdeveloped lungs, kidneys, brain, and cardiovascular system, which can have long-lasting effects on organ structure, metabolism, and susceptibility to chronic diseases later in life. Despite the numerous advantages of human milk, it is essential to recognize that it cannot completely reverse the permanent damage that may result from premature birth. Indeed, nutrition alone cannot thoroughly overcome these obstacles, regardless of the quality of human milk and its functional components.

Further studies are required to develop individualized nutritional strategies concerning sex differences between males and females, birthweight, birthweight small for gestational age, impaired placental flows, intrauterine and extrauterine growth restriction, and other comorbidities (such as respiratory distress, patent ductus arteriosus, bronchopulmonary dysplasia, necrotizing enterocolitis). Furthermore, novel opportunities such as webinars can support education around the growth and nutrition of preterm infants and remove differences in future outcomes among different centres.

**Figure 1:**
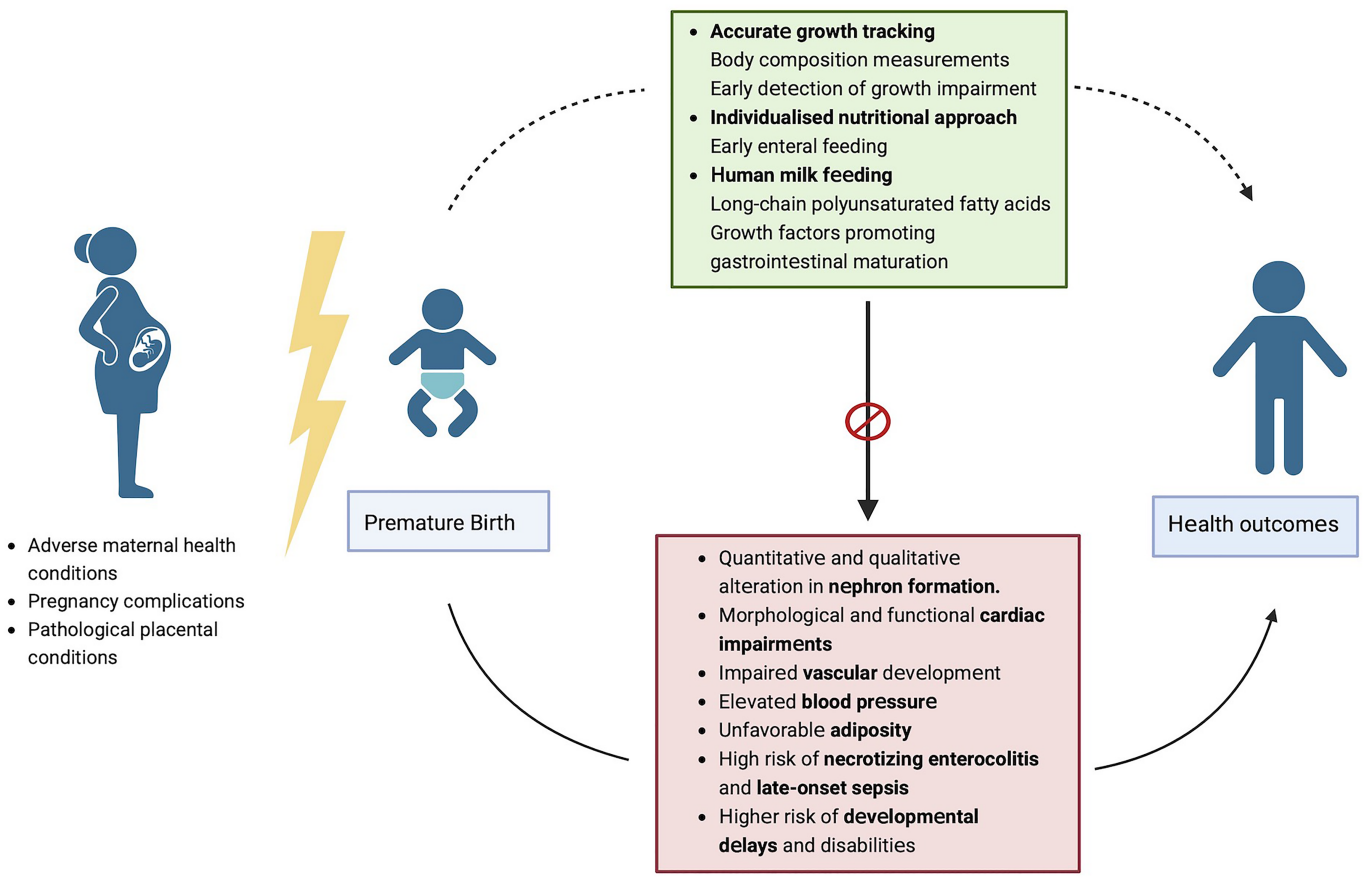
Strategies to modulate potential poor outcomes associated with prematurity. Figure created with Biorender.

We firmly believe that adequate nutritional intake and an individualized approach using human milk feeding are crucial to the health and development of preterm infants (Figure 1). Human milk remains an essential tool for supporting the health and development of premature infants, as it provides a multitude of benefits that can mitigate some of the adverse outcomes associated with premature birth. By feeding preterm infants with human milk, healthcare professionals and parents can support their growth, immune function, cognitive development, and overall well-being against all morbidities related to premature birth, thereby giving them the best possible start in life.

## Data Availability

The original contributions presented in the study are included in the article, further inquiries can be directed to the corresponding author.
